# The synergy of nutritional risk and iron deficiency as a predictor of mortality in older emergency department patients

**DOI:** 10.3389/fmed.2026.1769541

**Published:** 2026-07-21

**Authors:** Chao Zhang, Shijia Li, Ziyi Li, Xinhua He

**Affiliations:** 1 Department of Emergency Medicine, Beijing HuaiRou Hospital, Beijing, China; 2 Department of Pulmonary and Critical Care Medicine, Beijing HuaiRou Hospital, Beijing, China; 3 Beijing Key Laboratory of Cardiopulmonary-Cerebral Resuscitation Innovation and Translation, Beijing Chaoyang Hospital Emergency Medical Center Affiliated to Capital Medical University, Beijing, China

**Keywords:** elderly, emergency department, hemoglobin, iron deficiency, mortality, nutritional risk

## Abstract

**Background:**

Older patients in the emergency department (ED) often present with coexisting nutritional risk and iron deficiency (ID). However, this combined risk is frequently underrecognized and inadequately addressed in clinical practice.

**Objective:**

To investigate the independent and combined predictive value of nutritional risk and ID for 28-day mortality in older ED patients, and to clarify the underlying pathway-specifically, examining whether hemoglobin (Hb) acts as a mediator or whether direct effects are present.

**Methods:**

This prospective cohort study enrolled 443 patients aged ≥ 60 years from the ED resuscitation room. Patients were stratified into four groups based on their modified Nutrition Risk in the Critically Ill (mNUTRIC) score and ID status: G1, mNUTRIC < 5 without ID; G2, mNUTRIC < 5 with ID; G3, mNUTRIC ≥ 5 without ID; G4, mNUTRIC ≥ 5 with ID. Multivariate Cox regression was used to identify independent risk factors for mortality. Mediation analysis was performed to explore potential mechanistic pathways.

**Results:**

The overall 28-day mortality rate was 22.8%. Patients with both high nutritional risk and ID (G4) had the highest mortality (42.7%) and a risk 4.9 times greater than the G1 (adjusted HR = 4.901, 95% CI: 2.861–8.394, *p* < 0.001). Hb was a strong independent protective factor (HR = 0.976, *p* < 0.001). Crucially, the excess mortality risk in G4 was almost entirely concentrated in patients with concurrent anemia (HR = 6.224), but was absent in non-anemic patients (interaction *p* < 0.001). The combination of mNUTRIC score, Hb, and ID provided the best prediction of 28-day mortality (AUC = 0.779).

**Conclusion:**

The coexistence of high nutritional risk and ID exerts a powerful synergistic effect on mortality in older ED patients. Although associated with hemoglobin levels, this synergy appears to be primarily direct, suggesting that mechanisms beyond anemia contribute to the excess mortality. Integrated assessment of this “risk triad” may help identify high-risk patients earlier.

## 1 Introduction

Older patients in the critical care setting often experience functional decline, which is associated with significantly worse clinical outcomes compared to younger populations (1, 2). Among this vulnerable group, malnutrition has emerged as a key modifiable risk factor. Studies indicate that the prevalence of malnutrition or its risk in critically ill older adults can be as high as 50%, and is strongly associated with adverse outcomes such as infection, functional decline, and mortality (3, 4).

Within the spectrum of malnutrition, micronutrient deficiencies have received relatively less attention, with iron deficiency (ID) being a prime example. ID is the most common micronutrient deficiency in older adults and has complex etiologies, including inadequate dietary intake, malabsorption, chronic blood loss, and underlying chronic diseases (5, 6, 7).

In the context of emergency critical care, traditional nutritional assessment tools like Body Mass Index (BMI) are often unreliable due to fluid imbalances. The modified Nutrition Risk in the Critically Ill (mNUTRIC) score was developed to address this limitation by integrating factors such as age, Acute Physiology and Chronic Health Evaluation II (APACHE II) score, and Sequential Organ Failure Assessment (SOFA) score. This tool more accurately identifies patients with markedly increased nutritional needs, impaired nutrient utilization, and poor prognosis due to hypercatabolism and high inflammatory burden (8, 9). Notably, systemic inflammation triggered by nutritional risk further regulates hepcidin, imposing a “dual blockade” on systemic iron metabolism: it reduces iron absorption from the gut and blocks iron recycling from macrophages. This results in significantly reduced circulating iron availability, despite potentially adequate body iron stores, failing to meet physiological demands (10, 11). This suggests that high nutritional risk and ID may frequently coexist in critically ill patients and could exert synergistic adverse effects on prognosis.

Although previous studies have independently established associations of either nutritional risk or ID with poor outcomes in older patients (12, 13), the broader prognostic impact of ID has been demonstrated across diverse populations. In the general population, ID affects over 50% of individuals and is independently associated with increased long-term mortality (14). Similarly, in patients with myocardial infarction, ID is present in approximately 58% of patients and predicts a more than two-fold increased risk of all-cause mortality (15). In the surgical setting, preoperative ID has been shown to increase the risk of postoperative mortality by more than three-fold, along with higher rates of serious adverse events (16). Despite this accumulating evidence, the interaction between these two factors and the specific physiological pathways through which they collectively increase mortality risk remain unclear. Anemia, as the most direct and common clinical manifestation of both ID and malnutrition, impairs tissue oxygen delivery and accelerates organ failure (17, 18), and thus may play a central mediating role in this relationship. However, no study to date has systematically validated this complete clinical pathway—from “upstream risk” to “downstream decompensation”—in a high-risk population of older ED patients.

Therefore, this prospective cohort study was designed to comprehensively investigate the complex interplay between nutritional risk, ID, and 28-day mortality among older ED patients. We hypothesized that the co-occurrence of high nutritional risk and ID would exhibit a synergistic detrimental effect, resulting in the poorest clinical outcomes. Furthermore, we aimed to explore the potential mediating role of hemoglobin reduction in this association, while also considering the contribution of additional direct pathophysiological mechanisms.

## 2 Materials and methods

### 2.1 Study design and participants

This prospective observational cohort study was conducted in the ED resuscitation room of Beijing Chao-yang Hospital between September 2024 and March 2025. The inclusion criteria were: (1) age ≥ 60 years; and (2) admission to the ED resuscitation room. The following exclusion criteria were applied: (1) age < 60 years; (2) death within 24 h of admission; (3) receipt of intravenous or oral iron supplementation within the past month; (4) history of blood transfusion within the past 3 months; (5) cachexia (defined as BMI < 18.5 kg/m^2^ with > 5% weight loss in 3 months); (6) refusal to participate; (7) incomplete data. The study was approved by the Institutional Ethics Committee of Beijing Chao-yang Hospital (Approval No.: 2024-Ke-785), and written informed consent was obtained from all participants or their legal representatives.

### 2.2 Data collection and definitions

Upon enrollment, comprehensive demographic data (age, sex, body mass index) and pre-existing comorbidities (hypertension, diabetes, coronary heart disease, chronic kidney disease, stroke, cancer, heart failure, chronic obstructive pulmonary disease) were collected. Fasting venous blood samples were drawn within 48 h of admission for laboratory tests. The following parameters were measured: hemoglobin (Hb), platelet count (PLT), white blood cell count (WBC), serum creatinine (Scr), alanine aminotransferase (ALT), aspartate aminotransferase (AST), serum albumin (ALB), folate (FOL), and vitamin B12 (VitB12), serum ferritin (SF), serum iron (SI), and transferrin saturation (TSAT; calculated as SI/total iron-binding capacity ×100%). Given the characteristics of older patients in the ED resuscitation room, who often present with acute exacerbations of chronic conditions and a high incidence of acute cardiovascular events, ID was defined as serum ferritin < 100 μg/L or 100 ≤ serum ferritin < 300 μg/L with transferrin saturation (TSAT) < 20% (19). Anemia was defined using World Health Organization criteria: Hb < 130 g/L for men and < 120 g/L for women (20). High nutritional risk was defined as an mNUTRIC score ≥ 5 (21). Disease severity was assessed using APACHE II and SOFA scores. Nutritional risk was evaluated using the mNUTRIC score and the Nutrition Risk Screening 2002 (NRS2002) tool. Based on mNUTRIC score and iron status, patients were categorized into four groups: Group 1 (G1): low nutritional risk (mNUTRIC < 5) without ID; Group 2 (G2): low nutritional risk (mNUTRIC < 5) with ID; Group 3 (G3): high nutritional risk (mNUTRIC ≥ 5) without ID; Group 4 (G4): high nutritional risk (mNUTRIC ≥ 5) with ID.

### 2.3 Study outcome

The primary outcome measure was all-cause mortality within 28 days of admission to the ED resuscitation room. Survival status at 28 days was confirmed through telephone follow-up or review of electronic medical records.

### 2.4 Statistical analysis

Statistical analyses were performed using IBM SPSS Statistics (Version 25.0). The Shapiro–Wilk test was used to assess the normality of continuous variables. Normally distributed data are presented as mean ± standard deviation and compared using Student’s *t*-test or one-way analysis of variance (ANOVA). Non-normally distributed data are presented as median (interquartile range) and compared using the Mann–Whitney U test or Kruskal-Wallis H test. Categorical data are presented as numbers (percentages) and compared using the chi-square test. Kaplan–Meier curves were plotted for the four risk groups, and differences were compared using the log-rank test. Variables considered clinically relevant were included in a multivariate Cox proportional-hazards regression model to identify independent risk factors associated with 28-day mortality. Results are expressed as hazard ratios (HR) with corresponding 95% confidence intervals (CI). The predictive performance of Hb, mNUTRIC score, and iron status for 28-day mortality was evaluated by calculating the area under the receiver operating characteristic curve (AUC). Differences between AUCs were compared using DeLong’s test. A two-sided *p* value < 0.05 was considered statistically significant. Mediation analysis was performed using the PROCESS macro for SPSS (version 2.16.3) (22). Model 4 with 5,000 bootstrap samples was applied to generate bias-corrected 95% CIs for indirect effects. An indirect effect was considered significant if the 95% bootstrap CI did not include zero.

## 3 Results

### 3.1 Study population

**Figure 1:**
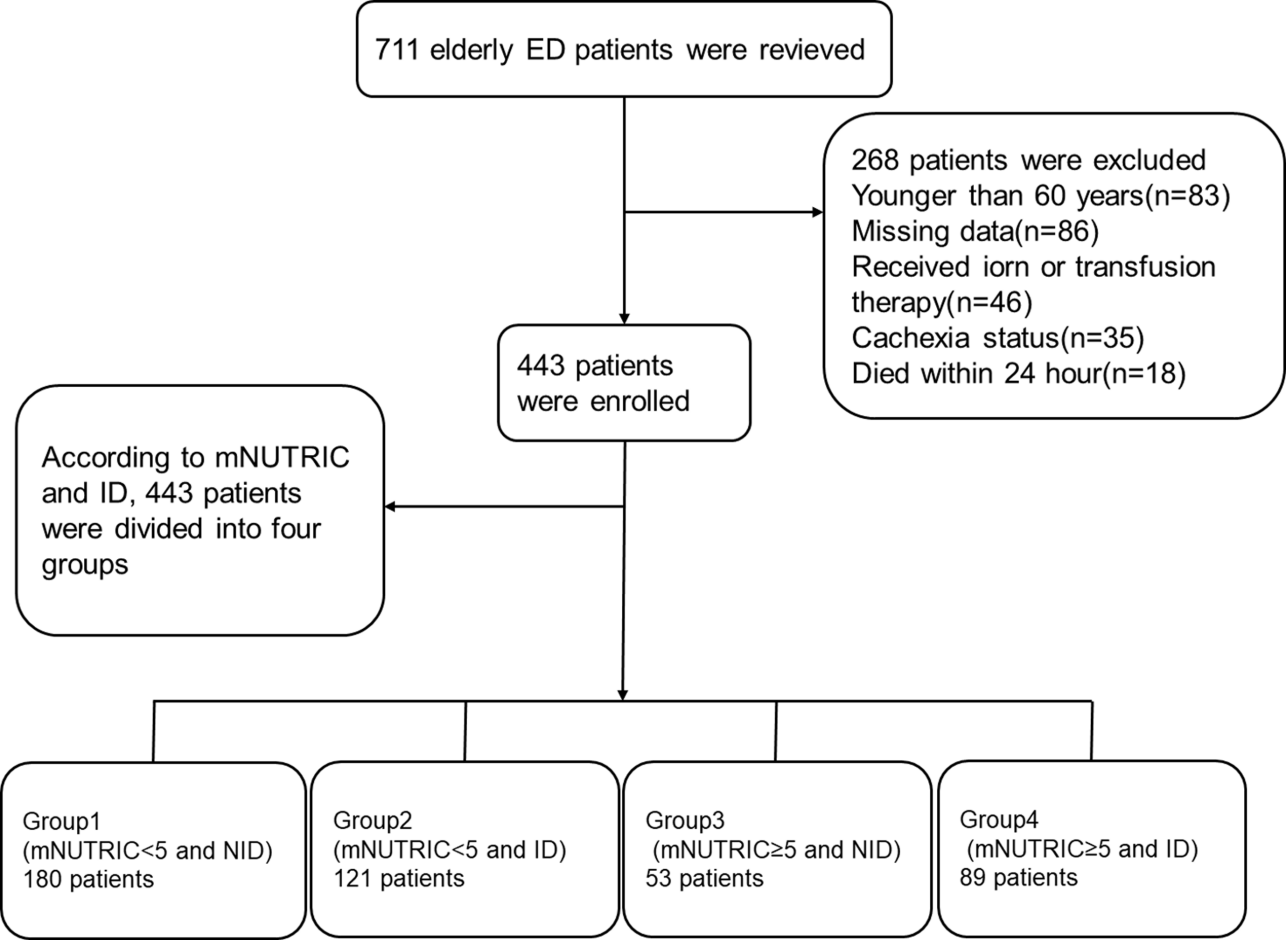
Patient selection flowchart.

A total of 711 older patients from the ED resuscitation room were initially assessed. After excluding 268 patients (83 aged <60 years, 86 with incomplete data, 46 receiving iron or transfusion therapy, 35 with cachexia, 18 who died within 24 h), 443 patients were included in the final analysis (Figure 1).

### 3.2 Baseline characteristics of patients by nutritional risk and ID status

The final cohort comprised 443 critically ill patients with a median age of 76.0 years (IQR: 68.0–84.0), of whom 264 (59.6%) were male. The median BMI was 23.4 kg/m^2^ (IQR: 22.0–25.0). The most prevalent comorbidities were hypertension (64.1%), anemia (51.7%), and diabetes mellitus (34.9%). According to mNUTRIC score and iron status, patients were divided into four distinct groups: G1 (*n* = 180), G2 (*n* = 121), G3 (*n* = 53), and G4 (*n* = 89). Baseline characteristics are detailed in Table 1. Significant differences among the four groups were observed in sex distribution, prevalence of chronic kidney disease (CKD), and key laboratory parameters including platelet count, serum creatinine, albumin, hemoglobin, serum ferritin, serum iron, folate, and vitamin B12 levels (all *p* < 0.05). Disease severity scores (APACHE II, SOFA) and nutritional risk scores (mNUTRIC, NRS2002) also differed significantly across groups (all *p* < 0.001). Notably, G4 exhibited the highest proportion of females (52.8%), the most severe disease scores (APACHE II: 19; SOFA: 5), lower hemoglobin levels (120.0 g/L), and the most pronounced indicators of ID (median serum iron: 4.4 μg/dL).

**Table 1:** Baseline characteristics by nutritional risk and ID status.

Variable	Overall (*n* = 443)	G1 (*n* = 180)	G2 (*n* = 121)	G3 (*n* = 53)	G4 (*n* = 89)	*p* value
Age (years)	76.0 (68.0–84.0)	74.0 (68.0–84.0)	75.0 (68.0–83.0)	78.0 (69.5–84.0)	79.0 (70.0–86.0)	0.135
Gender	443					0.038
Male, *n* (%)	264 (59.6%)	119 (66.1%)	71 (57.7%)	32 (60.4%)	42 (47.2%)	
Female, *n* (%)	179 (40.4%)	61 (33.9%)	50 (41.3%)	21 (39.6%)	47 (52.8%)	
BMI (kg/m^2^)	23.4 (22.0–25.0)	23.0 (21.5–24.7)	23.4 (22.0–24.8)	23.4 (22.5–25.0)	23.5 (21.5–25.4)	0.324
HTN, *n* (%)	284 (64.1%)	106 (58.9%)	82 (67.8%)	35 (66.0%)	61 (68.5%)	0.288
DM, *n* (%)	151 (34.1%)	60 (33.3%)	44 (36.4%)	15 (28.3%)	32 (36.0%)	0.790
CHD, *n* (%)	144 (32.5%)	57 (31.7%)	47 (38.8%)	16 (30.2%)	24 (27.0%)	0.291
CKD, *n* (%)	110 (24.8%)	28 (15.6%)	21 (17.4%)	35 (66.0%)	26 (29.2%)	<0.001
Stroke, *n* (%)	101 (22.8%)	39 (21.7%)	24 (19.8%)	16 (30.2%)	22 (24.7%)	0.432
Tumor, *n* (%)	73 (16.5%)	37 (20.6%)	13 (10.7%)	9 (17.0%)	14 (15.7%)	0.161
HF, *n* (%)	110 (24.8%)	46 (25.6%)	36 (29.8%)	13 (24.5%)	15 (16.9%)	0.184
COPD, *n* (%)	64 (14.4%)	24 (13.3%)	18 (14.9%)	5 (9.4%)	17 (19.1%)	0.454
Anemia, *n* (%)	229 (51.7%)	80 (44.4%)	72 (59.5%)	28 (53.8%)	49 (54.4%)	0.068
WBC (×10^9^/L)	9.6 (6.7, 13.3)	9.6 (6.7, 12.7)	8.5 (6.5, 12.4)	11.7 (6.3, 14.9)	10.0 (7.5, 15.5)	0.060
PLT (×10^9^/L)	182.0 (134.0–231.0)	181.0 (121.0–233.6)	189.0 (145.5–244.0)	153.0 (92.0–213.5)	184 (150.5–230.5)	0.017
Scr (μmol/L)	78.4 (56.0–132.0)	73.2 (52.6–100.9)	75.2 (62.4–121.8)	181.9 (92.9–403.5)	77 (53.3–175.5)	<0.001
ALT (U/L)	20.0 (12.00–38.0)	23.5 (13.0–42.8)	17.0 (11.0–31.5)	21.0 (11.5–49.0)	20.0 (12.0–30.5)	0.060
AST (U/L)	27.0 (19.0–45.0)	28.0 (19.0–49.0)	25.0 (18.5–37.5)	27.0 (17.5–81.5)	27.0 (19.0–40.5)	0.390
ALB (g/L)	33.60 ± 6.41	35.64 ± 5.73	31.83 ± 6.13	33.63 ± 7.11	31.89 ± 6.52	<0.001
Hb (g/L)	123.0 (96.0–142.0)	130.5 (97.3–155.0)	121.0 (97.5–135.5)	117.0 (90.0–146.5)	120.0 (96.0–132.5)	0.008
SF (μg/L)	215.8 (104.4–484.0)	383.5 (225.5–661.0)	92.2 (42.9–169.6)	647.8 (435.3–1131.9)	118.5 (63.3–185.4)	<0.001
SI (μg/dL)	6.0 (3.4–10.7)	8.6 (4.7–12.6)	5.3 (3.3–7.7)	6.9 (2.3–12.3)	4.4 (2.9–6.6)	<0.001
FOL (ng/mL)	7.24 (4.3–14.3)	6.1 (3.6–12.0)	7.5 (4.3–13.4)	8.6 (4.8–17.7)	8.6 (4.9–20.4)	0.044
VitB12 (pg/mL)	558.0 (356.0–1012.0)	720.5 (395.8–1211.0)	422.0 (310.5–640.5)	972.0 (483.5–1407.5)	471.0 (291.0–814.0)	<0.001
mNUTRIC (score)	4 (3–5)	3 (2–3)	4 (3–4)	6 (5–6.5)	6 (5–6)	<0.001
NRS2002 (score)	4 (3–4)	3 (2–4)	4 (3–4)	4 (3–4)	4 (4–5)	<0.001
APACHEII (score)	15 (12–19)	12 (9–16)	16 (14.0–19)	21 (16–25)	19 (14–22)	<0.001
SOFA (score)	5 (3–7)	4 (3–5)	4 (2–5)	7 (6–9.5)	5 (4–11)	<0.001
28-day mortality, *n* (%)	101 (22.8%)	21 (11.7%)	30 (24.8%)	12 (22.6%)	38 (42.7%)	<0.001

BMI, body mass index; HTN, hypertension; DM, diabetes mellitus; CHD, coronary heart disease; CKD, chronic kidney disease; HF, heart failure; COPD, chronic obstructive pulmonary disease; WBC, white blood cell count; PLT, platelet count; Scr, serum creatinine; ALT, alanine aminotransferase; AST, aspartate aminotransferase; ALB, albumin; Hb, hemoglobin; SF, serum ferritin; SI, serum iron; FOL, folate; VitB12, vitamin B12; APACHE II, Acute Physiology and Chronic Health Evaluation II; SOFA, Sequential Organ Failure Assessment.

### 3.3 Comparison between 28-day survivors and non-survivors

During the 28-day follow-up period, 101 patients died (mortality rate 22.8%). As shown in Table 2, non-survivors had a significantly higher proportion of females (46.5% vs. 36.5%, *p* = 0.002), CKD (32.7% vs. 22.5%, *p* = 0.038), anemia (75.2% vs. 44.7%, *p* < 0.001), and ID (67.3% vs. 41.5%, *p* < 0.001) compared to survivors. Non-survivors also presented with significantly higher serum creatinine, lower hemoglobin, lower serum ferritin, lower serum iron, and higher mNUTRIC, NRS2002, APACHE II, and SOFA scores (all *p* < 0.05).

**Table 2:** Baseline characteristics of the survivor and non-survivor groups.

Variable	Overall (*n* = 443)	Survival group (*n* = 342)	Non-survival group (*n* = 101)	*p* value
Age (years)	76.0 (68.0–84.0)	75.0 (68.0–84.0)	76.0 (68.5–85.0)	0.951
Gender, *n* (%)	443			0.002
Male, *n* (%)	264 (59.6%)	217 (63.5%)	47 (53.5%)	
Female, *n* (%)	179 (40.4%)	125 (36.5%)	54 (46.5%)	
HTN, *n* (%)	284 (64.1%)	214 (62.6%)	70 (69.3%)	0.215
DM, *n* (%)	151 (34.1%)	115 (33.6%)	36 (35.6%)	0.707
CHD, *n* (%)	144 (32.5%)	111 (32.5%)	33 (32.7%)	0.967
CKD, *n* (%)	110 (24.8%)	77 (22.5%)	33 (32.7%)	0.038
Stroke, *n* (%)	101 (22.8%)	72 (21.1%)	29 (28.7%)	0.107
Tumor, *n* (%)	73 (16.5%)	54 (15.8%)	19 (18.8%)	0.472
HF, *n* (%)	110 (24.8%)	85 (24.9%)	25 (24.8%)	0.983
COPD, *n* (%)	64 (14.4%)	54 (15.8%)	10 (9.9%)	0.139
Anemia, *n* (%)	229 (51.7%)	153 (44.7%)	76 (75.2%)	<0.001
ID, *n* (%)	210 (47.4%)	142 (41.5%)	68 (67.3%)	<0.001
WBC (×10^9^/L)	9.6 (6.7–13.3)	9.6 (6.6–13.1)	9.6 (6.7–15.0)	0.754
PLT (×10^9^/L)	182.0 (134.0–231.0)	188.0 (135.0–234.0)	169.0 (128.0–222.0)	0.299
Scr (μmol/L)	78.4 (56.0–132.0)	75.6 (55.0–126.5)	94.9 (63.5–203.1)	0.024
ALT (U/L)	20.0 (12.0–37.3)	20.0 (12.0–36.2)	19.0 (10.5–50.4)	0.995
AST (U/L)	27.0 (19.0–45.3)	27.0 (18.0–42.2)	30.0 (20.0–58.0)	0.147
BMI (kg/m^2^)	23.4 (22.0–25.0)	23.4 (22.0–25.0)	23.0 (21.7–25.0)	0.769
ALB (g/L)	33.60 ± 6.41	33.74 ± 6.15	33.13 ± 7.20	0.397
Hb (g/L)	123.0 (96.0–142.0)	130.5 (108.0–149.0)	94.0 (79.5–109.0)	<0.001
SF (μg/L)	215.8 (104.4–484.0)	236.6 (111.6–524.0)	167.2 (82.3–272.0)	0.001
SI (μg/dL)	6.0 (3.4–10.7)	6.6 (3.8–11.1)	5.2 (2.7–8.6)	0.004
FOL (ng/mL)	7.2 (4.3–14.3)	6.9 (4.0–13.4)	8.6 (4.9–18.7)	0.028
VitB12 (pg/mL)	558.0 (356.0–1012.0)	562.5 (361.0–1004.0)	538.0 (343.5–1023.5)	0.581
mNUTRIC (score)	4 (3–5)	3 (3–5)	4 (3–6)	<0.001
NRS2002 (score)	4 (3–4)	3 (3–4)	4 (3–5)	<0.001
APACHE II (score)	15 (12–19)	15 (11–19)	18 (14–23)	<0.001
SOFA (score)	5 (3–7)	4 (3–7)	5 (4–8)	<0.001

BMI, body mass index; HTN, hypertension; DM, diabetes mellitus; CHD, coronary heart disease; CKD, chronic kidney disease; HF, heart failure; COPD, chronic obstructive pulmonary disease; WBC, white blood cell count; PLT, platelet count; Scr, serum creatinine; ALT, alanine aminotransferase; AST, aspartate aminotransferase; ALB, albumin; Hb, hemoglobin; SF, serum ferritin; SI, serum iron; FOL, folate; VitB12, vitamin B12; APACHE II, Acute Physiology and Chronic Health Evaluation II; SOFA, Sequential Organ Failure Assessment.

### 3.4 Survival analysis

**Figure 2:**
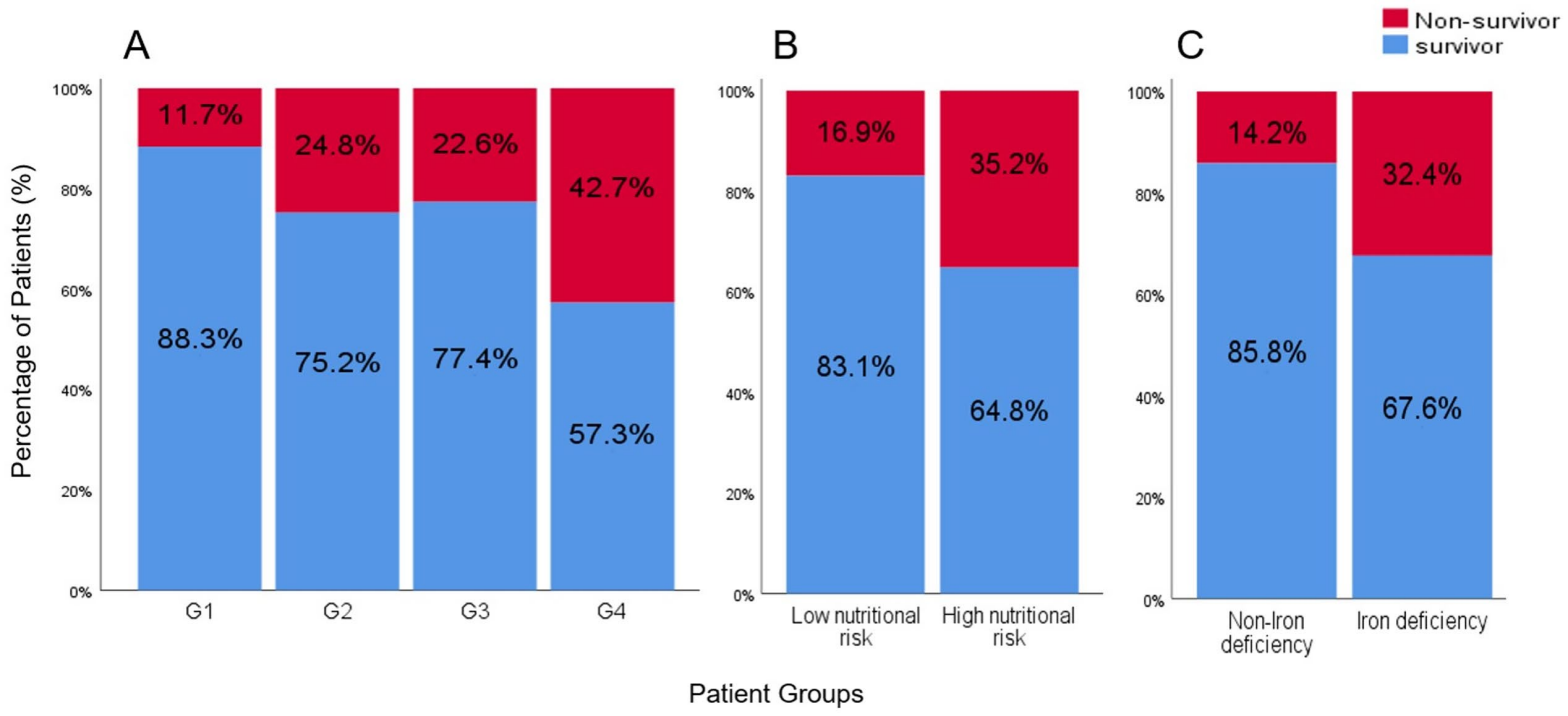
**(A)** Patients were stratified into four groups based on their mNUTRIC score and ID status: G1 (low nutritional risk, no ID), G2 (low nutritional risk, with ID), G3 (high nutritional risk, no ID), and G4 (high nutritional risk, with ID). **(B)** Patients were stratified into two groups based on their mNUTRIC score. **(C)** Patients were stratified into two groups based on their ID status. The 28-day mortality rate for each group is presented as a percentage.

The 28-day mortality rates for the four risk groups were as follows: G1:11.7% (21/180), G2: 24.8% (30/121), G3:22.6% (12/53), and G4:42.7% (38/89) (Figure 2A). Mortality in the low nutritional risk groups and high nutritional risk groups was 16.9% (51/301) and 35.2% (50/142) (Figure 2B). Mortality was 14.2% (33/233) in patients without ID and 32.4% (68/210) in those with ID (Figure 2C).

**Figure 3:**
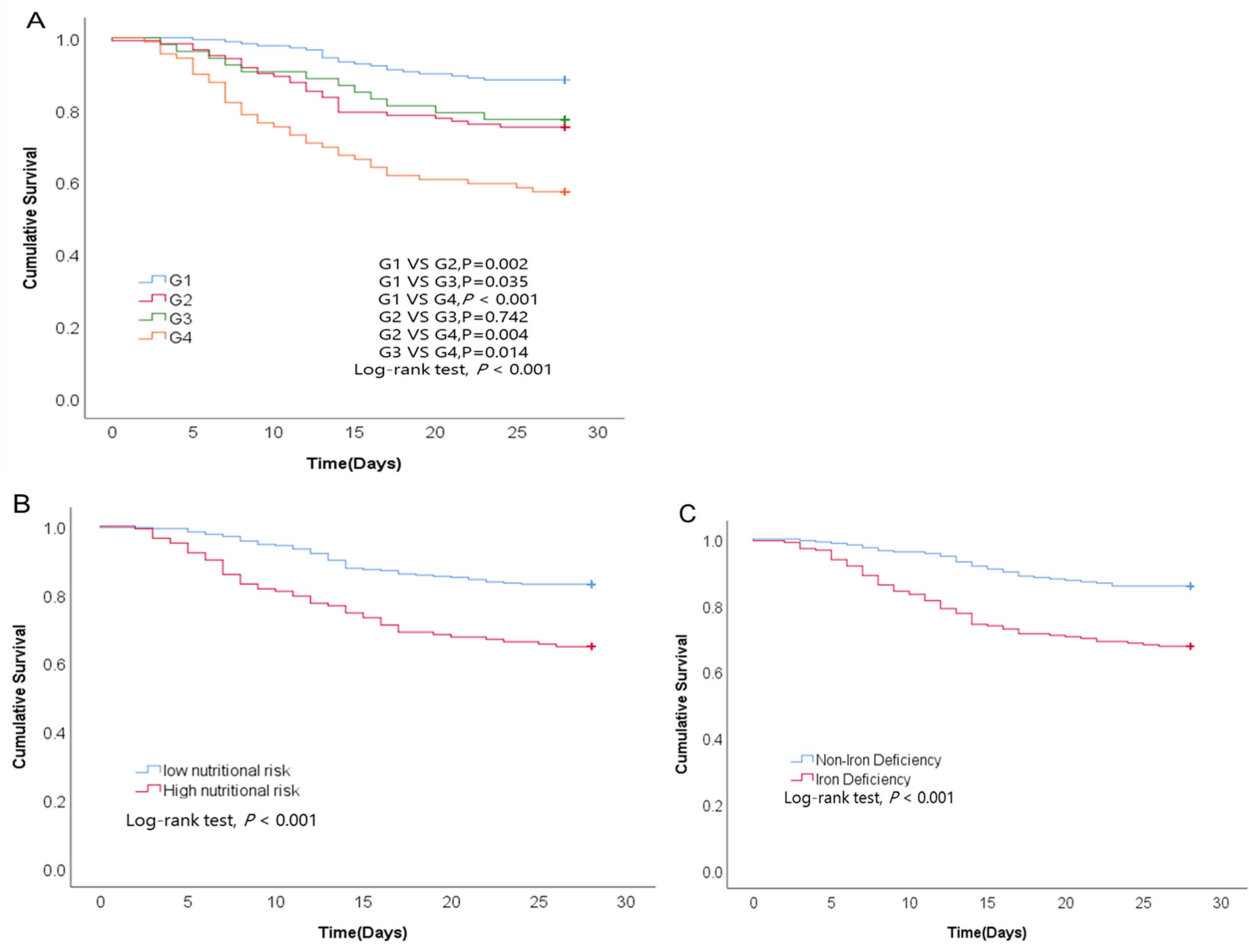
**(A)** Cumulative survival rate over 28 days is shown for the four patient groups (G1-G4). **(B)** Cumulative survival rate over 28 days is shown for the high nutritional risk and low nutritional risk groups. **(C)** Cumulative survival rate over 28 days is shown for the ID and non-ID groups.

Kaplan–Meier survival curves (Figure 3A) demonstrated significant differences in cumulative survival among the four groups (Log-rank *p* < 0.001). Pairwise comparisons indicated that survival in G4 was significantly lower than in G1 (Log-rank, *p* < 0.001), G2 (Log-rank, *p* = 0.004), and G3 (Log-rank, *p* = 0.014). G3 had a lower survival than G1 (Log-rank, *p* = 0.035), but no significant difference was found between G3 and G2 (Log-rank, *p* = 0.742). Survival in G2 was significantly lower than that in G1 (Log-rank, *p* = 0.002). Separate analyses confirmed that both high nutritional risk and ID alone were associated with significantly worse survival (Figures 3B,C, both Log-rank *p* < 0.001).

### 3.5 Multivariate Cox regression analysis for 28-day mortality

The independent association between the risk groups and 28-day mortality was assessed using multivariate Cox proportional hazards models (Table 3). In the unadjusted model (Model 1), all risk groups showed significantly higher mortality compared to the G1 reference group. Patients in the G2 had a 2.33-fold increased risk (HR = 2.332, 95% CI: 1.335–4.073, *p* = 0.003), while those in the G3 exhibited a 2.09-fold higher risk (HR = 2.085, 95% CI: 1.026–4.238, *p* = 0.042). Most notably, patients in the G4 demonstrated the highest mortality risk, which was 4.65 times greater than that of the G1 (HR = 4.649, 95% CI: 2.727–7.925, *p* < 0.001). After adjustment for demographic factors and comorbidities including age, gender, hypertension, diabetes, stroke, coronary heart disease, and heart failure (Model 2), the mortality risk remained significantly elevated in both the G2 (HR = 2.264, 95% CI: 1.295–3.958, *p* = 0.004) and the G4 (HR = 4.289, 95% CI: 2.502–7.354, *p* < 0.001). The association for the G3 was attenuated and became borderline significant (HR = 2.001, 95% CI: 0.983–4.072, *p* = 0.056). In the fully adjusted model (Model 3) that additionally included nutritional, laboratory, and clinical parameters (BMI, NRS2002, platelet count, hemoglobin, serum creatinine, albumin, vitamin B12, and folate), the synergistic effect of high nutritional risk combined with ID remained robust and statistically significant. G4 maintained a substantially elevated mortality risk (HR = 4.901, 95% CI: 2.861–8.394, *p* < 0.001). Similarly, G2 continued to show significantly higher mortality (HR = 2.229, 95% CI: 1.275–3.896, *p* = 0.005), while the G3 no longer demonstrated a statistically significant association (HR = 1.683, 95% CI: 0.825–3.433, *p* = 0.152). A significant trend of increasing mortality risk across the risk groups was observed in all three models (*p* for trend < 0.001 for all models). Hemoglobin was identified as a strong independent protective factor in Model 3 (HR = 0.976 per g/L increase, 95% CI: 0.966–0.986, *p* < 0.001).

**Table 3:** Cox proportional-hazards models for the association of risk groups with 28-day mortality.

Variables	Model 1			Model 2			Model 3		
	HR (95% CI)	*p*-value	*p* for trend	HR (95% CI)	*p*-value	*p* for trend	HR (95% CI)	*p*-value	*p* for trend
Risk group			<0.001			<0.001			<0.001
G1 group	Ref			Ref			Ref		
G2 group	2.332 (1.335–4.073)	0.003		2.264 (1.295–3.958)	0.004		2.229 (1.275–3.896)	0.005	
G3 group	2.085 (1.026–4.238)	0.042		2.001 (0.983–4.072)	0.056		1.683 (0.825–3.433)	0.152	
G4 group	4.649 (2.727–7.925)	<0.001		4.289 (2.502–7.354)	<0.001		4.901 (2.861–8.394)	<0.001	
Covariates									
Hb (g/L)	Not Applicable			Not Applicable			0.976 (0.966–0.986)	<0.001	

Model 1: unadjusted.

Model 2: adjusted for age, gender, hypertension, diabetes, Stroke, CHD, HF.

Model 3: adjusted for age, gender, hypertension, diabetes, Stroke, CHD, HF, BMI, NRS2002, PLT, Hb, Scr, ALB, VitB12, folate.

CHD, Coronary Heart Disease; HF, Heart Failure; PLT, Platelet Count; Scr, Serum Creatinine; BMI, body mass index; ALB, Serum Albumin; Hb, hemoglobin; VitB12, VitaminB12.

The outcome for all models is 28-day all-cause mortality.

### 3.6 Subgroup analysis of 28-day mortality

Subgroup analyses revealed significant effect modifications (Table 4). The association between G4 and mortality was markedly modified by anemia status (interaction *p* < 0.001). The excess risk was almost entirely concentrated in anemic patients (HR = 6.224, 95% CI: 3.320–11.665, *p* < 0.001) and absent in non-anemic patients (HR = 1.815, 95% CI: 0.576–5.719, *p* = 0.309). CKD status also showed significant effect modification (interaction *p* = 0.032), with the excess mortality risk in G4 present only in patients without CKD (HR = 3.288, 95% CI: 1.830–5.908, *p* < 0.001). No significant interactions were found for sex, age, or APACHE II score.

**Table 4:** Subgroup analysis of the association between high Nutritional Risk combined with ID (G4 vs. G1) and 28-day mortality risk.

Subgroup	Patients (*n*)	Deaths (*n*)	HR (95% CI)	*p* value	*p* for Interaction
Overall	443	101	4.649 (2.727, 7.925)	<0.001	
Sex					0.060
Male	264	47	6.631 (2.954, 14.888)	<0.001	
Female	179	54	2.891 (1.422, 5.879)	0.003	
Age					0.743
<76	221	48	6.330 (2.818, 14.218)	<0.001	
≥76	222	53	3.503 (1.723, 7.124)	0.001	
CKD					0.032
No	333	68	3.288 (1.830, 5.908)	<0.001	
Yes	110	33	1.147 (0.483, 2.696)	0.797	
Anemia					<0.001
No	214	25	1.815 (0.576, 5.719)	0.309	
Yes	229	76	6.224 (3.320, 11.665)	<0.001	
APACHE II score					0.248
<15	198	33	3.757 (1.605, 8.719)	0.002	
≥15	245	68	4.060 (1.989, 8.288)	0.001	

The hazard ratio (HR) for mortality in Group G4 is compared to the reference Group G1 across various patient subgroups. *p* for interaction tests the significance of the difference in HRs between subgroups. CKD, chronic kidney disease; APACHE II, Acute Physiology and Chronic Health Evaluation II.

### 3.7 Mediation role of hemoglobin

To elucidate the potential pathway through which nutritional risk and ID influence mortality, we conducted formal mediation analyses examining hemoglobin as a mediator. The results from both unadjusted and adjusted models are presented in Table 5. In the unadjusted model (Model 1), hemoglobin served as a significant mediator in the relationship between all risk groups and mortality compared to the G1 reference group. This was evidenced by statistically significant indirect effects, represented by the log-odds of mortality: G2 (effect = 0.28, 95% CI: 0.08–0.54), G3 (effect = 0.32, 95% CI: 0.00–0.70), and G4 (effect = 0.29, 95% CI: 0.09–0.58). The decomposition of these effects revealed that all risk groups showed significant negative associations with hemoglobin levels (path a: G2: *β* = −9.38; G3: *β* = −10.83; G4: *β* = −9.94; all *p* < 0.05), while lower hemoglobin levels were strongly associated with increased mortality risk (path b: OR = 0.97, *p* < 0.001). However, after adjustment for key clinical covariates (age, gender, creatinine, folate, vitamin B12, BMI, and albumin) in Model 2, the mediation pattern changed substantially. The indirect effects through hemoglobin were attenuated and no longer reached statistical significance for any risk group comparisons: G2 (effect = 0.22, 95% CI: −0.01–0.47), G3 (effect = 0.21, 95% CI: −0.13–0.62), and G4 (effect = 0.21, 95% CI: −0.01–0.47). This attenuation was primarily driven by the weakened associations between risk groups and hemoglobin levels (path a), with only G2 maintaining a statistically significant association (*β* = −7.36, *p* < 0.05) while G3 and G4 (*p* > 0.05) became non-significant. In contrast to the attenuated mediation effects, the direct effects of the risk groups on mortality remained robust and statistically significant after covariate adjustment. The G4 consistently demonstrated the strongest direct effect on mortality across both unadjusted (OR = 5.89, 95% CI: 3.04 to 11.42) and adjusted models (OR = 6.48, 95% CI: 3.27 to 12.84).

**Table 5:** Mediation analysis of hemoglobin.

Comparison (vs. G1)	Model	Path a: X → Hb β (95% CI)	Direct effect: X → Y OR (95% CI)	Indirect effect: X → Hb → Y Log-odds (95% CI)
G2	Model 1	−9.38 (−16.28, −2.48)*	2.40 (1.25, 4.59)*	0.28 (0.08, 0.54)*
	Model 2	−7.36 (−14.51, −0.20)*	2.64 (1.35, 5.18)*	0.22 (−0.01, 0.47)
G3	Model 1	−10.83 (−20.00, −1.66)*	1.74 (0.75, 4.06)	0.32 (0.00, 0.70)*
	Model 2	−7.03 (−16.37, 2.31)	1.81 (0.75, 4.39)	0.21 (−0.13, 0.62)
G4	Model 1	−9.94 (−17.54, −2.34)*	5.89 (3.04, 11.42)*	0.29 (0.09, 0.58)*
	Model 2	−7.00 (−14.78, 0.79)	6.48 (3.27, 12.84)*	0.21 (−0.01, 0.47)

Model 1 is unadjusted.

Model 2 is adjusted for age, gender, creatinine, folate, vitamin B12, body mass index, and albumin. Path a represents the unstandardized regression coefficient (β) from the linear regression model of the risk group (X) on hemoglobin (Hb, the mediator). Path b represents the log-odds from the logistic regression model of Hb on mortality risk (Y). Path b was identical in all models: OR = 0.97 (95% CI: 0.96–0.98, *p* < 0.001). The direct effect is the odds ratio (OR) of the risk group (X) on mortality (Y) after accounting for the mediator (Hb). The indirect effect (X → Hb → Y) is the product of Path a and Path b, representing the effect mediated through Hb, and is presented on the log-odds. It was tested using bootstrapping with 5,000 samples. CI, confidence interval.

*p < 0.05.

### 3.8 Predictive value of key parameters for 28-day mortality

**Figure 4:**
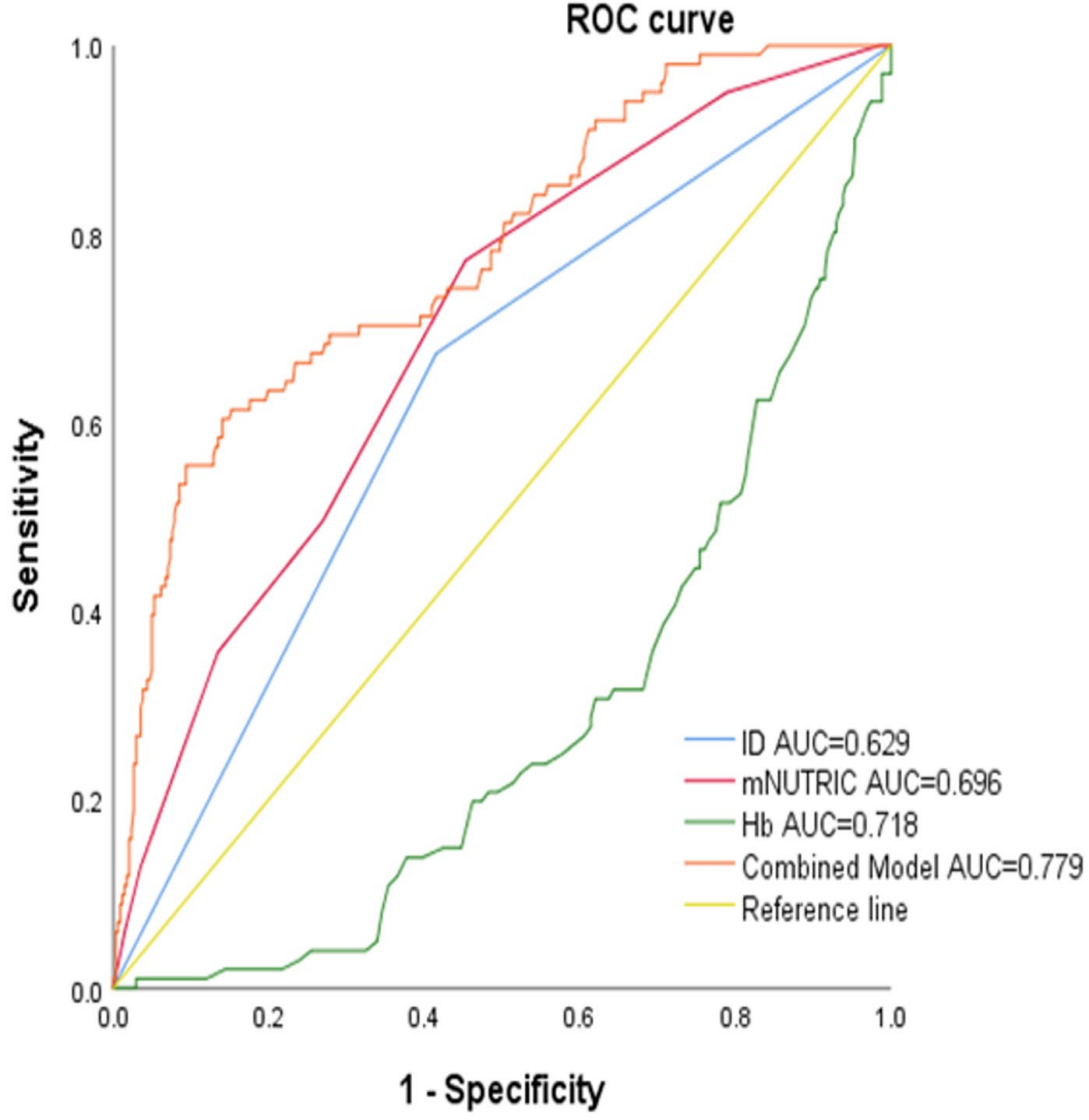
The predictive performance of the modified Nutrition Risk in the Critically Ill (mNUTRIC) score, hemoglobin (Hb) level, and iron deficiency (ID) status, both individually and in combination, for 28-day mortality is shown.

The predictive performance of key parameters was evaluated using ROC curve analysis (Figure 4). The AUCs for the mNUTRIC score, hemoglobin, and ID alone were 0.696, 0.718, and 0.629, respectively. Hemoglobin was a protective factor for 28-day mortality. The combination of all three parameters showed the highest predictive value, with an AUC of 0.779 (95% CI: 0.726–0.831), a sensitivity of 77.2%, and a specificity of 65.3%.

## 4 Discussion

This prospective cohort study of 443 older critically ill patients in the emergency department revealed that both high nutritional risk (32.1%) and ID (47.4%) are highly prevalent. Their co-occurrence exerts a significant synergistic effect on 28-day mortality risk (HR = 4.901). These findings provide a novel perspective for understanding the mechanisms underlying adverse outcomes in this complex patient population.

Our study confirms that high nutritional risk (mNUTRIC score ≥5) and ID are independent risk factors for mortality, aligning with previous research. For instance, Rahman et al. (23) pioneered the finding that the mNUTRIC score effectively identifies critically ill patients who benefit the most from nutritional support and are at the highest risk of mortality. Multiple prospective studies have demonstrated the strong predictive power of mNUTRIC for short-term mortality in ICU patients compared to other nutritional screening tools (24–26). Similarly, in heart failure patients, Jankowska et al. (6) established that ID is a powerful predictor of mortality, with detrimental effects independent of anemia. Lasocki S et al. (27), in a randomized controlled trial, found that intravenous iron supplementation for ID significantly reduced 90-day mortality and improved one-year survival in critically ill patients ready for discharge after prolonged hospitalization. Our study connects these independent lines of evidence and reveals that in the broader, more heterogeneous older ED population, “high nutritional risk” and “ID” interact synergistically, conferring a risk greater than the sum of their individual effects.

We propose that systemic inflammation may be the core pathophysiological link underpinning this synergy (28). Critical illness in the elderly inherently involves a state of high inflammation and hypercatabolism (29), with a high nutritional risk (reflected by an elevated mNUTRIC score) being a manifestation of this state. Previous studies have documented substantial protein loss (approximately 10–15% of initial total protein content) within the first 10 days of ICU admission despite adequate prior nutritional status and intake (30). This aligns with our observation of lower albumin levels in the high nutritional risk groups (G3, G4). The ensuing systemic inflammatory response significantly upregulates hepcidin expression, which inhibits both intestinal iron absorption and macrophage iron recycling. Concurrently, in senescent cells, impaired ferritinophagy sequesters iron within ferritin, reducing intracellular bioavailable iron and consequently decreasing circulating iron levels (31, 32). This aligns perfectly with the framework proposed by Pasricha et al. (11) who emphasized inflammation as a primary driver of acquired iron deficiency. Thus, patients in Group G4 are trapped in a vicious cycle: high inflammation drives nutritional deterioration and disrupts iron utilization, while ID, in turn, impairs mitochondrial function and cellular energy metabolism (33), exacerbating fatigue and sarcopenia, ultimately leading to the depletion of organ reserve capacity. Our findings are highly consistent with the “inflammation-ID” axis theory (10).

A pivotal finding of our study is the elucidation of hemoglobin’s role in this pathway. While Hb was a strong independent protective factor, our mediation analysis indicated that the synergistic mortality risk in G4 was primarily direct, not mediated through Hb. This crucial nuance suggests that the detrimental effects extend beyond the well-known consequences of anemia on tissue oxygen delivery (34–36). The excess risk was almost exclusively manifested in anemic patients, fitting the clinical pathway “Inflammation/High Nutritional Risk → Functional ID→ Anemia.” However, the persistence of a strong direct effect after accounting for Hb implies that the harm of this synergy also involves non-hematopoietic mechanisms. Iron is indispensable for cellular processes in immune function, muscle metabolism, and cardiac function. Its deficiency in these contexts, independent of anemia, can directly contribute to organ dysfunction and failure (37, 38).

Importantly, although our multivariate Cox regression analysis had already adjusted for major comorbidities including malignancies and CKD, the synergistic mortality risk of G4 remained robust and independent. Furthermore, the effect modification observed by CKD status in our subgroup analysis is also instructive. The association between G4 and mortality risk disappeared in patients with CKD. This may be because CKD patients are already in a state of intense inflammation and dysregulated iron metabolism, where mortality risk is primarily driven by renal function itself (39, 40). Consequently, the additional relative risk posed by our defined “high nutritional risk-ID” phenotype may be overshadowed. This suggests that this risk stratification approach may have greater discriminatory value in older ED patients without CKD.

Our findings have immediate clinical implications. The combination of the mNUTRIC score, iron status, and hemoglobin achieved superior predictive value (AUC = 0.779) for 28-day mortality compared to any single parameter. This provides a practical “risk triad” approach for rapidly identifying the most vulnerable older patients in the ED resuscitation room. Although correcting malnutrition and ID during the acute inflammatory phase is challenging, recent research in non-emergency fields offers insights. Early, protocolized nutritional therapy has been shown to improve outcomes in at-risk older adults (41). Moreover, interventional studies, such as these by Lasocki et al. (27), have demonstrated that correcting ID with intravenous iron in critically ill patients can improve survival, while a recent study found, in acute myocardial infarction patients with ID, demonstrate that correcting ID with intravenous iron can improve survival (42). Future interventional studies should specifically target this high-risk ‘risk triad’ population to determine whether early, combined nutritional and iron supplementation strategies can effectively break this vicious cycle and improve prognosis.

This study has several limitations. First, the single-center design may limit generalizability, necessitating validation by multi-center studies. Second, we used a composite definition for ID and did not directly measure biomarkers like hepcidin or IL-6, limiting a deeper exploration of the inflammation-iron metabolism axis. Third, the observational study design cannot definitively establish causality. Although we proposed a plausible mechanistic pathway, definitive causal conclusions await confirmation from prospective interventional studies.

## 5 Conclusion

In older emergency department patients, the synergy between high nutritional risk and ID independently predicts increased 28-day mortality, an effect mediated largely through non-anemic pathways. The combined “risk triad” of mNUTRIC score, iron status, and Hb enables early identification of high-risk individuals. These findings advocate for a paradigm shift toward combined nutritional and iron-metabolism interventions to improve survival in this vulnerable population.

## Data Availability

The raw data supporting the conclusions of this article will be made available by the authors, without undue reservation.
